# Utilization and Perceived Need for Caregiver Support Services: Implications for Emotional Wellbeing

**DOI:** 10.1093/geroni/igaf122.3476

**Published:** 2025-12-31

**Authors:** Tochukwu Okolie, Heather Menne

**Affiliations:** University of Connecticut, Mansfield Center, Connecticut, United States; Miami University, Oxford, Ohio, United States

## Abstract

The demonstrable efforts of family members providing care to an older person in the United States continue to make research headlines. Nonetheless, family caregivers providing hours of unpaid care are disproportionately at risk of declining emotional wellbeing. One of the protective mechanisms against this decline may be caregiver programs and services including: caregiver education/training, counseling, and support services. Hence, the aim of the study was to explore the differences in emotional wellbeing associated with: (i) attending caregiver support services, and (ii) a perceived need for caregiver support services. Data from the 2023 wave of the National Survey of Older Americans Act Participants (NSOAAP) was used (n = 1004). Univariate, bivariate, and regression techniques assessed the relationship between emotional wellbeing and the predictors of interest. The regression results show that compared to those who have attended counseling services, emotional wellbeing was about 0.17 points higher among those who have not attended, controlling for other variables in the model (β = 0.166, p = 0.004). Also, compared to those who perceived the need for counseling services, emotional wellbeing was about 0.3 points higher among those who do not, controlling for other variables in the model (β = 0.312, p < .0001). Also, being a male or a Black/African American were each significantly associated with an increase in emotional wellbeing, while accounting for other factors. The results suggest a need to understand the motivations and barriers to counseling services. There is also a need to sensitize caregivers on the need for these services.

